# The activity of cAMP-phosphodiesterase 4D7 (PDE4D7) is regulated by protein kinase A-dependent phosphorylation within its unique N-terminus

**DOI:** 10.1016/j.febslet.2015.02.004

**Published:** 2015-03-12

**Authors:** Ashleigh M. Byrne, Christina Elliott, Ralf Hoffmann, George S. Baillie

**Affiliations:** a Institute of Cardiovascular and Medical Sciences, College of Medical, Veterinary and Life Sciences, University of Glasgow, Glasgow G12 8QQ, UK; b Philips Research Eindhoven, Molecular Diagnostics, Eindhoven, The Netherlands

**Keywords:** PDE4D7, PKA, Cyclic-AMP, Peptide array, Prostate cancer

## Abstract

The cyclic AMP phosphodiesterases type 4 (PDE4s) are expressed in a cell specific manner, with intracellular targeting directed by unique N-terminal anchor domains. All long form PDE4s are phosphorylated and activated by PKA phosphorylation within their upstream conserved region 1 (UCR1). Here, we identify and characterise a novel PKA site (serine 42) within the N-terminal region of PDE4D7, an isoform whose activity is known to be important in prostate cancer progression and ischemic stroke. In contrast to the UCR1 site, PKA phosphorylation of the PDE4D7 N-terminus appears to occur constitutively and inhibits PDE4 activity to allow cAMP signalling under basal conditions.

## Highlights

• PDE4D7 is phosphorylated by PKA in the unique N-terminal region at serine 42.
• PDE4D7 phosphorylation at serine 42 negatively regulates PDE4 activity.
• Ablation of phosphorylation at serine 42 activates PDE4D7 and this reduces phosphorylation in the UCR1 domain.

## 1 Introduction

3′,5′-cyclic adenosine monophosphate (cAMP) is a ubiquitous intracellular second messenger that acts to orchestrate a number of important physiological functions that are triggered by activation of specific cell-surface receptors. Specificity of receptor action is often underpinned by the compartmentalisation of intermediates within the cAMP-signalling cascade. Discrete positioning of enzymes that synthesise cAMP (adenylate cyclase), are activated by cAMP (PKA, EPAC and cyclic nucleotide – gated ion channels) or degrade the second messenger (phosphodiesterases) allow the cell to tailor cellular responses following signals generated by a number of receptors coupled to Gαs [1]. The duration and strength of signals produced by cAMP effectors is often heavily influenced by action of a super-family of enzymes that has evolved to degrade cyclic-nucleotides, the phosphodiesterases (PDEs) [2].

Of particular interest is the PDE4 family of enzymes, which is made up of over 25 different isoforms, many of which have important, non-redundant functions [3]. Often, the function of a particular PDE4 isoform is conferred by its unique N-terminal, which acts as a “postcode” to anchor PDE4 enzymes to discrete intracellular domains where they sculpt signal-specific cAMP gradients. PDE4s also contain a catalytic unit and regulatory domains termed “upstream conserved regions one and two” (UCR1/2) which are highly conserved throughout the isoforms [4]. All long-form PDE4s contain UCR1, which contains a PKA motif that becomes phosphorylated during conditions of raised cAMP [5]. Such an action serves to activate PDE4 and rapidly reduce the local concentration of cAMP. This feedback loop underpins the transient nature of cAMP signals and ensures a rapid but fleeting response to activation of Gαs-coupled receptors [6].

In addition to phosphorylation of UCR1, the long isoform PDE4D3 undergoes PKA phosphorylation within its unique N terminus [5]. This modification does not affect activity but instead increases the affinity of binding to the A-kinase anchor protein, mAKAP [7]. To date, this is the only known case of a long PDE isoform being phosphorylated by PKA other than within its UCR1 domain. Using peptide array technology and a novel phospho-specific antibody, we demonstrate that PDE4D7, an isoform who’s activity is known to be important in prostate cancer progression [8] and ischemic stroke [9], is also phosphorylated by PKA within its unique N terminus on serine 42. We show modification of PDE4D7 in this way occurs under basal conditions, reduces PDE4D7 activity, and we hypothesise that this feature allows basal cAMP signalling, which may be necessary for cellular homeostasis and could be involved in the cAMP sensitive progression of prostate cancer from the androgen sensitive to androgen insensitive state.

## 2 Materials and methods

### 2.1 Reagents

Forskolin (Sigma) and KT5720 (Enzo) were dissolved in dimethyl sulfoxide. Anti-PKA phospho substrate (RXXpS) antibody was supplied from Cell Signalling, USA: Cat. No. 9621. Anti-phospho PDE4D7-serine42 antibody was custom made by AMSBIO (Europe) in rabbits against a phosphorylated peptide corresponding to residues ^34^EPYLVRRL(p)SCRN^45^. Total PDE4D7 antibody was custom made by Altabioscience (UK) against a GST-fusion of the whole unique N terminal region of PDE4D7.

### 2.2 Peptide array

Peptide libraries were produced by automatic SPOT synthesis and synthesized on continuous cellulose membrane supports on Whatman 50 cellulose membranes using Fmoc-chemistry with the AutoSpot-Robot ASS 222 (Intavis Bioanalytical Instruments AG, Köln, Germany) as previously described by us [10]. PKA phosphorylation of an immobilized library of PDE4D7 peptides was undertaken using 100 units of purified PKA catalytic subunit (Promega). Recombinant kinase was diluted in phosphorylation buffer (20 mM Tris–HCl; pH 7.5, 10 mM MgCl_2_, 0.5 mM CaCl_2_, 1 mM DTT, 0.2 mg/ml BSA, 1 mM ATP) and incubated with arrays at 30 °C for 30 min with shaking.

### 2.3 Site directed mutagenesis of PDE4D7

Site-directed mutagenesis was performed using the Quickchange kit (Stratagene) according to manufacturer’s instructions. The following primers were used to create the required full length and N terminal mutants. PDE4D7 S42A mutant, forward primer: AGACTTGCATGTCGCAATATTCAGCTTCCCCCTCTC, reverse primer: ATTGCGACATGCAAGTCTCCGGACAAGATAGGGTTCCATTCC. S42D mutant, forward primer: CGGAGACTTGACTGTCGCAATATTCAGCTTCCCCCTCTC, reverse primer: GGGATAGAACAGGCCTCTGAACTGACAGCGTTATAAGTCG.

### 2.4 Purification of GST-PDE4D7

Briefly; BL21 cells were transformed with the fusion protein and induction was brought about by addition of 1 mM of isopropyl-β-d-thiogalactopyranoside (IPTG). Following protein induction, cells were lysed by sonication and lysate was incubated with Glutathione Sepharose beads (Amersham). Following an incubation period, the beads were washed in elution buffer (50 mM Tris pH 8.0) + glutathione (6.8 mg of reduced glutathione (Sigma) per 1 ml of elution buffer). The beads were pelleted by brief centrifugation and the eluate was collected. Overnight dialysis of the collected eluate was carried out in dialysis buffer (100 mM NaCl, 50 mM Tris–HCl; pH 8.0, 5% glycerol, 5 mM DTT) in slide-a-lyzer cassettes (Piercenet) at 4 °C to remove any detergents or glutathione. Following recovery of the eluates from the cassettes, the protein concentration was determined by a Bradford assay.

### 2.5 In vitro PKA phosphorylation of PDE4D7

**Fig. 1:**
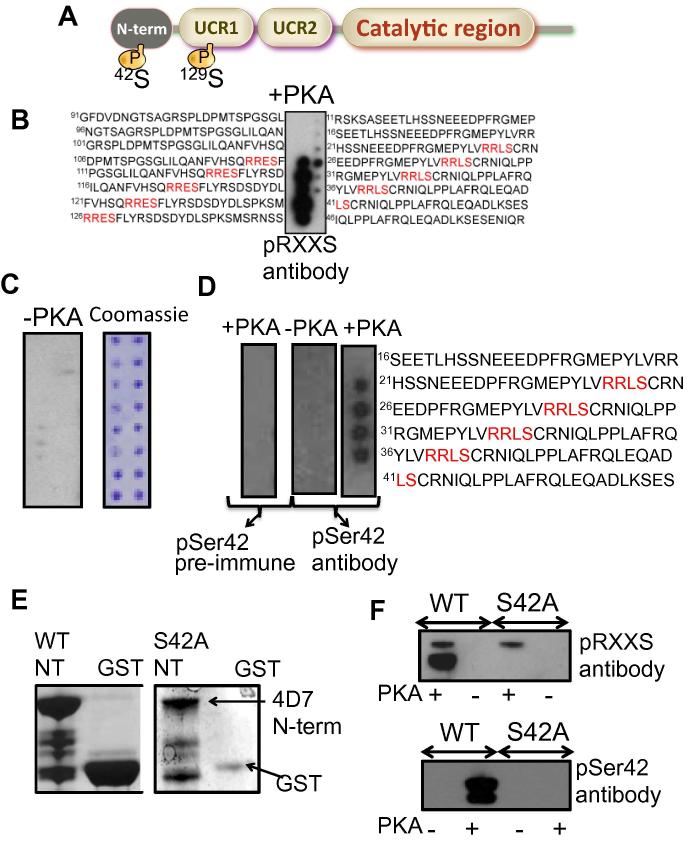
(A) Schematic depicting the domain structure of PDE4D7 including isoform specific N-terminal region (N-term), upstream conserved regions 1 and 2 (UCR1/2) and the catalytic region. (B) Peptide arrays containing immobilised peptides corresponding to the regions of the PDE4D7 N-terminal and UCR1 were subjected to phosphorylation following incubation with the PKA catalytic subunit. Phosphorylation (dark spots) was detected using a phospho-PKA substrate antibody. (C) Controls for the experiment described in B. The experiment was conducted without the catalytic subunit of PKA (left panel) and the presence of peptides was detected using Coomassie (right panel). (D) Validation of a novel Serine 42 phospho-specific antibody. The serine 42 phospho-specific antibody (right panel) or pre-immune serum (left panel) was used to detect phosphorylated peptides on peptide arrays corresponding to the PDE4D7 N-terminal. An experimental control conducted without the catalytic subunit of PKA was also undertaken (middle panel). (E) GST-fusion constructs (wild type and S42A) corresponding to the N-terminal region of PDE4D7 were purified and (F) used in a phosphorylation assay conducted with the catalytic subunit of PKA. The phosphorylation of GST-fusion constructs was detected using the serine 42 phospho-specific antibody or phospho-PKA substrate antibody. All data in Fig. 1 typical of *n* = 3.

Purified (2 μg) wt GST-PDE4D7 unique N terminal region and S42A wt GST-PDE4D7 unique N terminal region (Fig. 1E/F) or VSV-pull downs of Wt, S42A and S42D transfections (Fig. 3C) were incubated with 25 units of purified PKA catalytic subunit (Promega) in phosphorylation buffer (20 mM Tris–HCl; pH 7.5, 10 mM MgCl_2_, 0.5 mM CaCl_2_, 1 mM DTT, 0.2 mg/ml BSA, 1 mM ATP) for 1 h at 30 °C with gentle agitation. The samples were run on an SDS–PAGE gel and immunoblotted with the PKA phospho-substrate and phospho-specific antibodies.

### 2.6 Cellular transfection of wt and mutant PDE4D7 constructs and cell treatments

VSV tagged wild type PDE4D7, S42A PDE4D7, S42D PDE4D7 and dnPDE4D7 were transfected into HEK293 cells using Polyfect transfection reagent (Qiagen). Cells were treated with KT5720 (4 μM) for 20 min prior to forskolin (100 μM) treatment for the time points indicated, or forskolin alone (100 μM). Control cells were treated with DMSO or left untreated (NT). Cell lysates were harvested in KHEM buffer (50 mM HEPES pH 7.4, 50 mM KCL, 1.92 mM MgCl2) (for PDE activity assays) or 3T3 lysis buffer (25 mM HEPES, 10% w/v glycerol, 50 mM NaCl, 1% w/v Triton × 100, 50 mM NaF, 30 mM NaPP, 5 mM EDTA, pH7.4) containing Complete, EDTA-free Protease Inhibitor Cocktail Tablets (Roche) and PhosStop Phosphatase Inhibitor Cocktail Tablets (Roche).

### 2.7 Phosphodiesterase activity assays

Phosphodiesterase activity was measured using a radioactive cAMP hydrolysis assay that has been described previously [5]. [8-^3^H] adenosine cyclic-3′,5′-mono-phosphate was sourced from Amersham Biosciences (Little Chalfont, UK) and cyclic-3′,5′-mono-phosphate from Sigma. The substrate concentration used for PDE assays was 150 nM, and the specific PDE activity was determined as pmol cAMP hydrolysed/min/mg protein. PDE activities were then normalised for expression of construct, and the data were normalised to untreated (NT) wild type PDE4D7 activity.

## 3 Results

Sequence analysis of the PDE4D7 sequence uncovered a novel, putative consensus PKA site in the unique N-terminal region (^37^LVRRLSCR^44^) in addition to the already known site in UCR1 (^125^QRRESFL^131^) (Fig. 1A). To determine whether the 4D7 N-terminal motif is a PKA substrate, immobilised peptide array libraries of the N-terminal and UCR1 regions were synthesised using peptide array. Peptide array has been used by our group to successfully identify ubiquitination [11] and SUMOylation [12] sites on PDE4D5, and PKA sites on PDE8 [13], PI3K [14] and DNAPK [15]. Peptide arrays of overlapping 25-mer peptides, sequentially shifted by 5 amino acids and spanning the PDE4D7 N-terminal and UCR1 regions were incubated with a PKA assay mix before detection of phosphorylation using a PKA phospho-substrate antibody (Fig. 1B). Only two regions of phosphorylation were detected and these contained the previously known PKA site in UCR1 (^125^QRRESF^130^) and the new site in the unique N-terminal (^38^VRRLSC^43^) (Fig. 1B). No phosphorylation was detected when active PKA was omitted from the assay mix (Fig. 1C left panel) and peptide spotting was verified by coomassie staining (Fig. 1C right panel). In addition to the PKA phospho-substrate antibody, phosphorylation of serine 42 by PKA on peptide array was also be detected by a novel phospho-specific antibody raised against a peptide containing a phosphorylated version of the 4D7 N-terminal PKA site. (Fig. 1D, right panel). No such phosphorylation was detected by pre-immune serum or when PKA catalytic subunit was omitted from the assay mix (Fig. 1D left and middle panels).

Wild type N-terminal GST-PDE4D7 (WT-NT) and mutant N-terminal GST-PDE4D7-S42A (S42A-NT) were purified (Fig. 1E) and incubated with a PKA assay mix containing active purified PKA catalytic unit. Phosphorylation of the serine 42 site in the wild type N-terminal construct was detected by the PKA phospho-substrate antibody and our phospho-specific S42 antibody (Fig. 1F). However, neither picked up phosphorylation in the S42A mutants (Fig. 1F). Gratifyingly, the PKA phospho-substrate antibody also detected auto-phosphorylation of the PKA catalytic unit (Fig. 1F, upper panel, upper band), whereas phospho-specific S42 antibody did not.

**Fig. 2:**
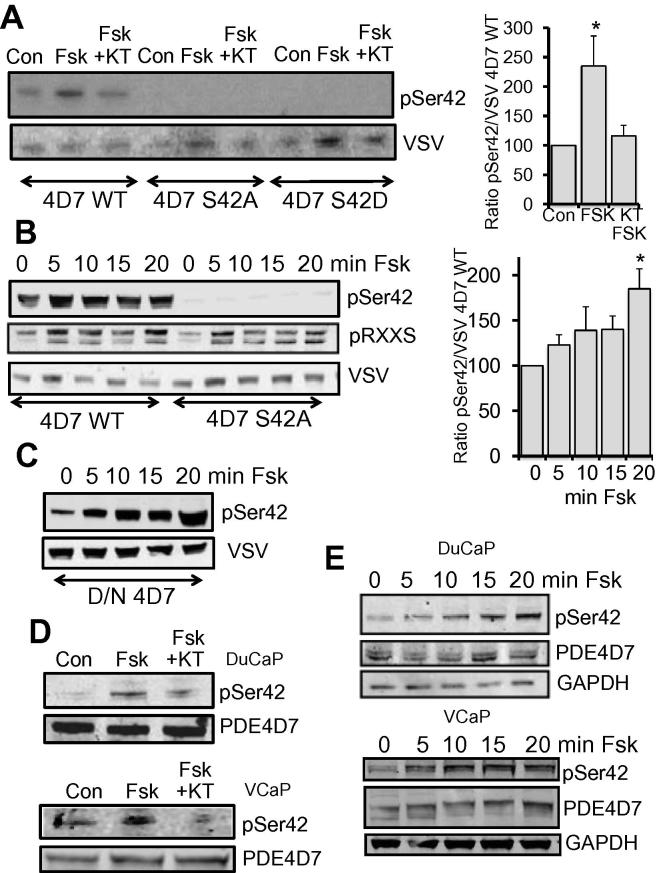
(A) HEK293 cells were transfected with VSV-tagged PDE4D7 wild type or the S42A or S42D mutants and treated with Forskolin or Forskolin after a pretreatment with the PKA inhibitor KT5720. Cellular lysates were blotted with serine 42 phospho-specific antibody or VSV antibody. Quantifications on right panel, ^∗^ = *P* < 0.05. (B) HEK293 cells were transfected with VSV-tagged PDE4D7 wild type or the S42A mutant and treated with Forskolin over 20 min. Cellular lysates were blotted with serine 42 phospho-specific antibody or phospho-PKA substrate antibody or VSV antibody. Quantifications on right panel, ^∗^ = *P* < 0.05. (C) HEK293 cells were transfected with VSV-tagged PDE4D7 wild type or the dominant negative, catalytically inactive mutant (D/N) and treated with Forskolin over 20 min. Cellular lysates were blotted with serine 42 phospho-specific antibody or VSV antibody. (D) DuCaP and VCaP cells and treated with Forskolin or Forskolin after a pretreatment with the PKA inhibitor KT5720. Cellular lysates were blotted with Serine 24 phospho-specific antibody orPDE4D7 antibody. (E) DuCaP and VCaP cells were treated with Forskolin over 20 min. Cellular lysates were blotted with serine 42 phospho-specific antibody or PDE4D7 antibody or GAPDH antibody. All data in Fig. 2 typical of *n* = 3.

To determine whether PKA phosphorylation of PDE4D7 at serine 42 could occur in a cellular context, HEK293 cells were transfected with VSV-tagged constructs of wild type PDE4D7 and the mutants S42A and S42D. Cells were left untreated or pre-treated with the PKA inhibitor KT5720, before endogenous cAMP levels were elevated using the adenylate cyclase activator forskolin. Cell lysates were then probed with our phospho-specific S42 antibody and an antibody against VSV to evaluate protein loading and construct expression (Fig. 2A). Basal levels of S42 phosphorylation could be detected and these were significantly increased following forskolin treatment. The forskolin-induced increase in phosphorylation was attenuated in KT pre-treated cells, indicating that PKA was the kinase responsible (Fig. 2A). No phosphorylation of PDE4D7 S42 was detected in cells expressing the S42A or S42D mutant. A forskolin time course showed that S42 phosphorylation steadily increased up to 20 min (Fig. 2B, upper panel and right panel). As expected, no phosphorylation of the S42A PDE4D7 mutant could be detected. To show that forskolin was active throughout the time course, PKA phosphorylation of non-specified substrates could be seen using the PKA phospho-substrate antibody (Fig. 2B, middle panel). Interestingly, a mutant of PDE4D7 carrying a single substitution that renders the enzyme completely inactive, resulted in a more robust and sustained phosphorylation of S42 (Fig. 2C). Presumably, this is because the “dead” enzyme has lost the ability to influence local cAMP concentration around it, leading to unfettered phosphorylation by PKA. As PDE4D7 activity is significantly down-regulated between androgen sensitive and androgen insensitive prostate cancer, and has been shown to mediate androgen sensitive prostate cancer cell proliferation [8], we were keen to determine if the phosphorylation of PDE4D7 could be observed in the androgen sensitive prostate cancer cell lines DuCaP and VCaP. Basal S42 phosphorylation was detected in both lines and this was increased following forskolin treatment and blocked by KT5720 (Fig. 2D). In a manner consistent with that seen for overexpressed PDE4D7 (Fig. 2B), endogenous PDE4D7 in DuCaP (Fig. 2E, upper panel) and VCaP cells (Fig. 2E, lower panel) became phosphorylated on S42 gradually following forskolin treatment (Fig. 2E).

**Fig. 3:**
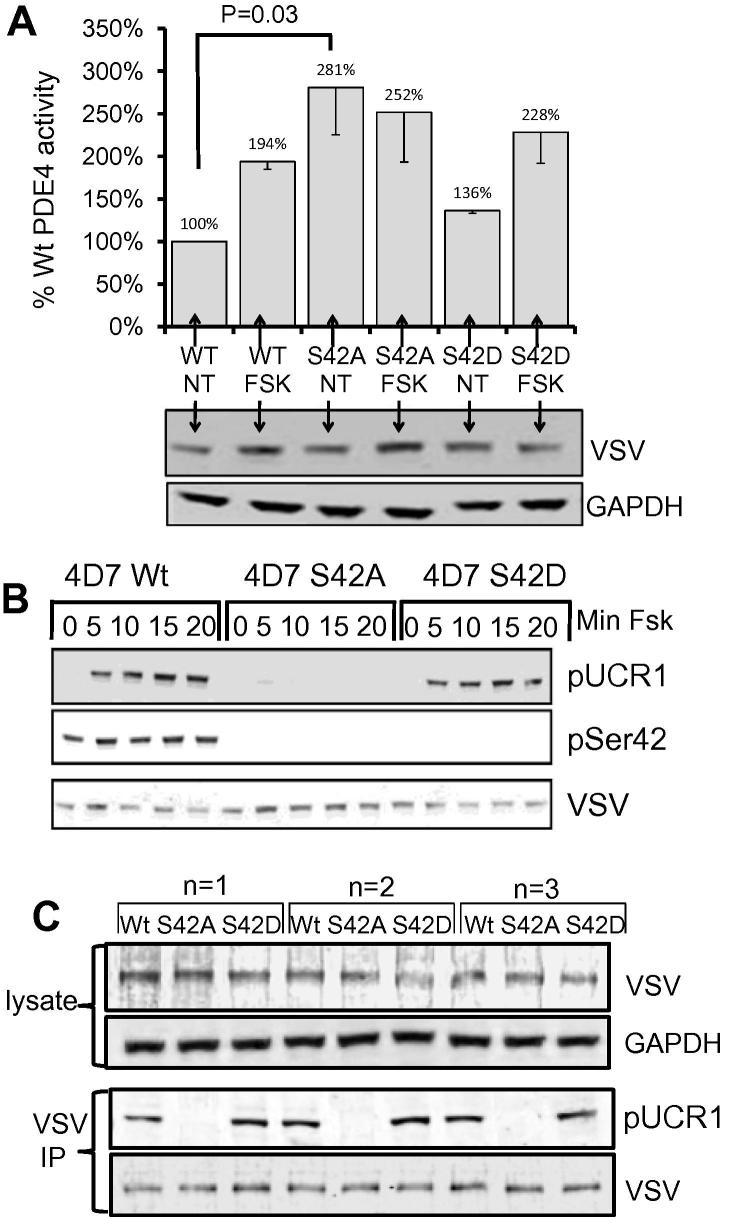
(A) HEK293 cells were transfected with VSV-tagged PDE4D7 wild type or the S42A or S42D mutants and treated with Forskolin. Cellular lysates were blotted for VSV or GAPDH. The PDE activity of cellular lysates was determined and expressed as a percentage of wild type control. ANOVA was used to determine significance of change (*n* = 3). (B) HEK293 cells were transfected with VSV-tagged PDE4D7 wild type or the S42A or S42D mutants and treated with Forskolin over a 20 min time course. Cellular lysates were blotted with serine 42 phospho-specific antibody or VSV antibody or a phospho-UCR1 (S129) antibody. Data typical of *n* = 3. (C) VSV-tagged PDE4D7 wild type or the S42A or S42D mutants were pulled down from transfected HEK293 and subjected to phosphorylation by PKA catalytic unit. Ips were blotted with a phospho-UCR1 (S129) antibody or VSV. Data for each of three replicates is shown.

As all long form PDE4 isozymes are activated following PKA phosphorylation of their UCR1 domain [5], creating a feedback loop to bring about cessation of cAMP signalling events, we were interested to see if N-terminal phosphorylation affected PDE4D7 activity. Lysates isolated from HEK293 cells, which had been transfected with VSV-tagged WT or mutant PDE4D7 constructs, were assayed for PDE activity, following 5 min forskolin challenge. Transfection efficiency was evaluated by Western blotting (Fig. 3A, lower panel). As expected, WT PDE4D7 doubled in activity after forskolin treatment (Fig. 3A, bar chart). Interestingly, the phospho-resistant S42A mutant exhibited an active phenotype with increased basal activity compared to wtPDE4D7 of >170% (*p* = 0.03, ANOVA) and forskolin challenge did not further increase its activity (Fig. 3A, bar chart). The phospho-mimetic S42D mutant, on the other hand, showed a small non-significant increase in basal activity compared to WT PDE4D7 NT (36%). The activity of this mutant was increased to a similar extent as WT PDE4D7 following forskolin challenge. We suggest that the hyperactivity associated with the S42A mutant is a result of the fact that phosphorylation of this site provides a mode of negative regulation for PDE4D7, ablation of which leads to activation. It is noteworthy that this site is basally phosphorylated in all of the cell lines investigated here, implying that the cell may require an inactive form of PDE4D7 for normal cellular homeostasis. We were unable to recreate phosphorylation-dependent enzyme inhibition at this site with the phospho-mimic mutant S42D. It behaved in a similar way to WT PDE4D7 under both basal conditions and forskolin treatment (Fig. 3A, bar chart), suggesting that the negative charge introduced by the substitution was not sufficient to evoke a functional change.

Since ablation of Ser42 phosphorylation led to a more active form of PDE4D7, we decided to determine whether this would impinge on the phospho-dynamics of the UCR1 site. Lysates prepared from cells overexpressing WT PDE4D7 and the S42A, S42D mutants were immunoblotted for phospho-UCR1 and phospho-Ser42. In support of the notion that blockade of the S42 phospho-site increases enzymatic activity, very little phosphorylation of UCR1 could be detected in the S42A mutant, even after 20 min forskolin treatment (Fig. 3B, upper panel). Presumably, the increase in PDE4 activity conferred by this mutation, acts to diminish local cAMP concentrations, in turn, attenuating PKA activity and preventing phosphorylation at the UCR1 PKA site, though it is possible that S42A directly affects the phosphorylation of UCR1 by PKA as isolated VSV Ips of the S42A mutant did not get phosphorylated by active PKA catalytic subunit, whereas wild type and S42D mutant did (Fig. 3C).

As with activity measurements, the WT PDE4D7 and S42D mutant behaved in a similar fashion with respect to UCR1 phosphorylation suggesting that the substitution of a negatively charged amino acid at S42 did not mimic the phosphorylation. In both cases (WT PDE4D7 and S42D mutant), no basal UCR1 phosphorylation could be detected, with a sustained phosphorylation being triggered by forskolin.

## 4 Discussion

Fine control of compartmentalised cAMP signalling is underpinned by the discrete positioning of phosphodiesterase enzymes which act to both maintain basal cAMP concentrations and shape cAMP gradients following activation of cell surface receptors [1]. Aberrant signalling within the cAMP signalling system has been closely linked with prostate cancer progression [16]. Changes in adenylate cyclase activity [17], PKA catalytic subunit expression [18] and most recently PDE4 expression [8] have all been observed between androgen-sensitive and androgen independent cancer phenotypes. In the latter case, transcripts from three sub-families (PDE4A, PDE4B and PDE4D) were detected, with PDE4D being the most highly expressed. Separation of a range of prostate cancer models and xenografts into androgen sensitive and androgen independent categories showed that PDE4D isoforms were downregulated in the androgen independent prostate cancer models. PDE4 isoform profiling identified PDE4D7 as the most important PDE4 isoform in the regulation of prostate cancer growth with high expression in androgen sensitive cells and a dramatic decline into androgen insensitivity [8]. Inhibition of a PDE4D7 pool sequestered to a sub-plasma membrane compartment increased prostate cancer cell proliferation and PDE4D7 has now been proposed as a novel biomarker for diagnosis of the AI prostate cancer phenotype. Investigation into the mechanism behind PDE4D7 expression changes in prostate cancer cell lines demonstrated that transcription from the PDE4D7 locus was not directly controlled by the androgen receptor [8] and further studies are required to elucidate the molecular events leading to changes in cellular PDE4D7 levels.

In this paper we provide evidence of a novel PKA phosphorylation site on PDE4D7 that alters its activity. Blockade of the phosphorylation of PDE4D7 on serine 42 dramatically increases its activity suggesting that phosphorylation at this site serves to inhibit the enzyme. Interestingly, the phosphorylation of PDE4D7 on serine 42 can be detected in the prostate cancer cell lines DuCaP and VCaP opening up the possibility that this post-translational modification may further promote the proliferative signalling observed following a reduction in PDE4D7 activity. Presumably, the increases in cAMP caused by this event could contribute to the AI phenotype by over exciting the autocrine and paracrine signalling systems that support androgen receptor transactivation [19]. More specifically, it is likely that membrane bound PDE4D7 regulates the cAMP gradients that are formed following activation of various Gas coupled receptors, which are known to signal partly through transactivation of the androgen receptor [20]. Recent reports of PDE4B knockdown following oxidative stress challenge have also been shown to promote growth of castration-resistant prostate cancer cells [21]. Experiments using our phospho-serine 42 specific antibody to screen AS and AI cell lines and xenografts may shed more light on the possible importance of PDE4D7 phosphorylation as an contributing event in the molecular pathology of prostate cancer.
